# Identification of benthic diatoms isolated from the eastern tidal flats of the Yellow Sea: Comparison between morphological and molecular approaches

**DOI:** 10.1371/journal.pone.0179422

**Published:** 2017-06-16

**Authors:** Sung Min An, Dong Han Choi, Jung Ho Lee, Howon Lee, Jae Hoon Noh

**Affiliations:** 1 Marine Ecosystem and Biological Research Center, Korea Institute of Ocean Science & Technology, Ansan, Republic of Korea; 2 Department of Marine Biology, University of Science and Technology, Daejeon, Republic of Korea; 3 Department of Biology Education, Daegu University, Gyeongsan, Republic of Korea; National Cheng Kung University, TAIWAN

## Abstract

Benthic diatoms isolated from tidal flats in the west coast of Korea were identified through both traditional morphological method and molecular phylogenetic method for methodological comparison. For the molecular phylogenetic analyses, we sequenced the 18S rRNA and the ribulose bisphosphate carboxylase large subunit coding gene, *rbcL*. Further, the comparative analysis allowed for the assessment of the suitability as a genetic marker for identification of closely related benthic diatom species and as potential barcode gene. Based on the traditional morphological identification system, the 61 isolated strains were classified into 52 previously known taxa from 13 genera. However, 17 strains could not be classified as known species by morphological analyses, suggesting a hidden diversity of benthic diatoms. The Blast search on NCBI’s Genebank indicated that the reference sequences for most of the species were absent for the benthic diatoms. Of the two genetic markers, the *rbcL* genes were more divergent than the 18S rRNA genes. Furthermore, a long branch attraction artefact was found in the 18S rRNA phylogeny. These results suggest that the *rbcL* gene is a more appropriate genetic marker for identification and classification of benthic diatoms. Considering their high diversity and simple shapes, and thus the difficulty associated with morphological classification of benthic diatoms, a molecular approach could provide a relatively easy and reliable classification system. However, this study suggests that more effort should be made to construct a reliable database containing polyphasic taxonomic data for diatom classification.

## Introduction

Diatoms are the most dominant taxa among the various microalgae and are known to account for ca. 40% of the total primary production in the ocean [1, 2]. Diatoms also play an important role in the biogeochemical cycles of carbon and silica [3]. In tidal flats, especially, benthic diatoms are the most dominant and diverse group and are key organisms that contribute to the preservation of the ecological functions of tidal flats such as primary production, nutrient cycling, and sediment stabilization [4–7]. Thus, the ecology and diversity of diatoms in tidal flats has received attention for a long time [8–11]. Although the study of diatom diversity has a relatively long history, overcoming the limitations of morphological classifications remains to be problematic. The small size and simple forms of benthic diatoms have made it difficult to study their diversity [12–14]. Furthermore, since the classification system is based on morphological characteristics of the type specimen, it is difficult to determine whether species having a similar form that appear in a variety of environments are the same species or different ones.

Since molecular techniques were applied to diatom research for the first time in the 1980s [15], molecular phylogenetic studies have been widely performed to identify and classify diatoms to overcome morphological limitations [16–19]. DNA barcoding is a method for α-taxonomy using molecular analyses based on differences in DNA sequences according to species. Therefore, unique DNA sequences can be referred to as tags or barcodes for each taxon [20]. Using DNA barcoding techniques, even morphologically similar strains can be identified at the species level. These molecular phylogenetic analyses have also enabled the rapid, convenient, and accurate classification of diatoms and have thus contributed considerably to studies on the diversity of diatoms.

Specific marker genes are used for molecular phylogenetic analyses. Different DNA regions within the nuclear rRNA gene, as well as mitochondrial and chloroplast genes, have been used for the phylogenetic analysis of diatoms [21]. Among them, nuclear 18S rRNA has been the most widely used [20, 22, 23]. The ribulose-1,5-bisphosphate carboxylase large subunit (*rbcL*) gene in chloroplasts has also been used for the phylogenetic study of diatoms [16, 24–26]. In addition, the cytochrome c oxidase subunit I (*coxI*), internal transcribed spacer (ITS), and ribulose-1,5-bisphosphate carboxylase small subunit (*rbcS*) have been used for the phylogenetic study of diatoms [16, 21, 27, 28]. However, these genetic makers have fewer records in public databases compared with the 18S rRNA gene.

In this study, morphological and molecular taxonomic characteristics of benthic diatoms isolated from tidal flats were investigated to evaluate the applicability of molecular phylogenetic approaches using 18S rRNA and *rbcL* genes. In addition, we present morphological as well as genetic information on the benthic diatoms. Although this research does not reveal the complete diversity of diatoms in tidal flats, it will be helpful in further studies on the diversity of benthic diatoms in various environments throughout the world.

## Materials and methods

### Collection, isolation and development of new strains

**Fig 1:**
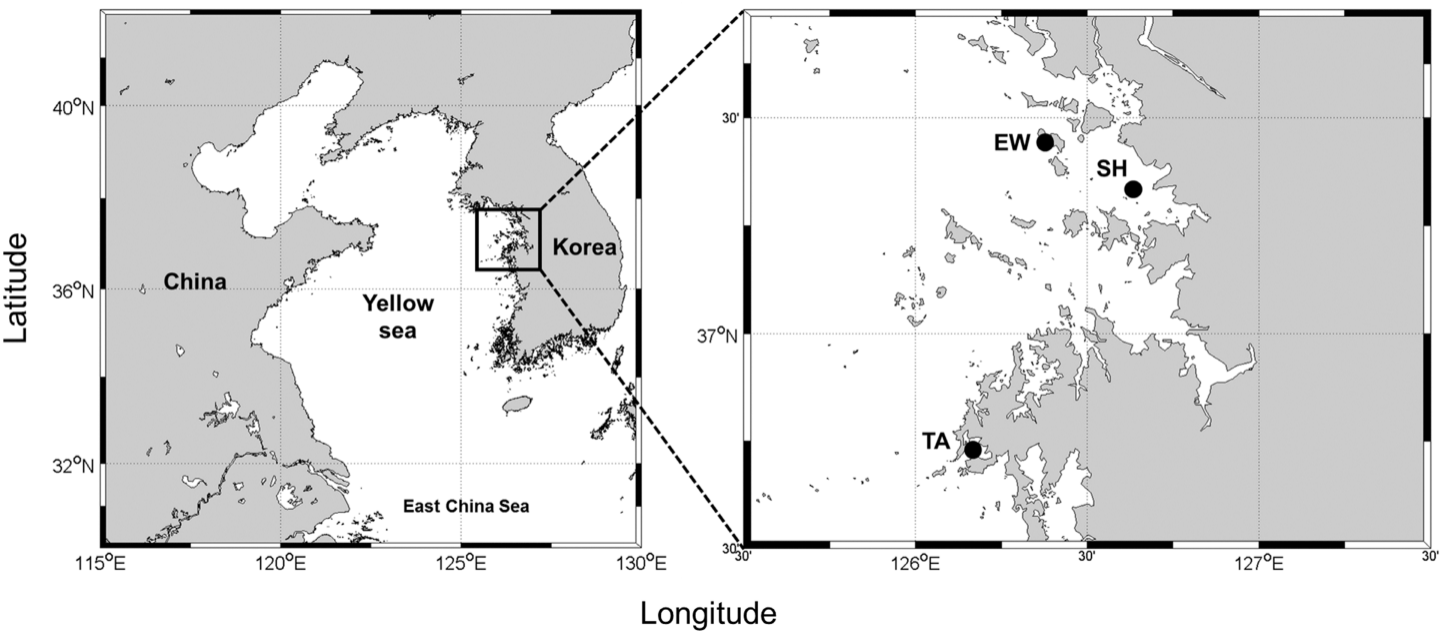
Map of the study sampling locations. (TA: Taean, SH: Sihwa, EW: Eulwang-ri).

Benthic diatoms were collected mainly from tidal flats of Geunso Bay in Taean (36° 44' 12.06" N 126° 10' 47.52" E), Eulwang-ri (37° 26' 43.67" N 126° 22' 18.07" E), and saline Sihwa (37° 18' 46.73" N 126° 36' 32.64" E) along the west coast of Korea (Fig 1). The numbers of strains obtained in each region were 53 in Geunso Bay and four in Sihwa, and four in Eulwang-ri. Most samples were obtained in the Geunso Bay where regular monthly surveys had been conducted from 2009. Geunso Bay is a semi-enclosed bay with an area of 87 km^2^, and the water depth at high tide is 2–4 m depending on the area. There is no inflow river, and facies are predominantly sandy silt. The Oi tidal flat, where Sihwa station is located, has an area of 0.025 km^2^, and the facies are predominantly silty sand. Eulwang-ri is a sandy facies and there is a beach near the sampling station.

To obtain sediment samples containing diatoms, the surface of the tidal flat was scratched to a depth of ca. 2 mm and the sediment collected in a conical tube. Samples were transported to the laboratory under refrigerated conditions and then incubated at ± 2°C of the *in situ* temperature. Diatom strains were isolated within 1 day of sampling. A single-diatom cell was isolated under an inverted microscope (Eclipse Ti-U; Nikon, Tokyo, Japan) using a glass Pasteur pipette and placed into a 24-well plate containing f/2 medium with silicate (Sigma-Aldrich, St. Louis, MO, USA). After confirmation of monoclonal growth, each culture was transferred to a new tissue culture flask (Falcon, Cockeysville, MD, USA) containing 35 ml of fresh medium for one week. Several cultures suspected to be a mixture were further isolated by a dilution method [29]. All strains were incubated at 15°C under a 12:12 h light-dark cycle. Illumination was provided by a fluorescent lamp with an irradiance of ca. 100 μmol photons m^-2^s^-1^. The strains were transferred to fresh medium every 2 or 3 weeks. Research activities at the sampling areas of this study did not require specific permission because the areas are not restricted or ecosystem protected. Endangered and protected species do not live in the study area and thus were not included in the survey.

### Morphological observations

Monoclonal cultures of benthic diatom strains were identified to the genus or species level by morphological features based on observations under light and scanning electron microscopy. For the light microscopy examination, diatom cultures were treated with acid to prepare cleaned frustules [30], and then permanent slides were made using Mountmedia (Wako Pure Chemical Industries, Osaka, Japan). The slides were examined using light microscopy under a ×100 oil immersion objective lens (Eclipse 80i; Nikon). For scanning electron microscopy examination, diatom cells fixed with Lugol’s solution were filtered onto a polycarbonate filter (diameter of 25 mm; pore size of 1 or 2 μm) and then washed with distilled water. The filter papers were dehydrated in a graded ethanol series (10%, 25%, 50%, 75%, 90%, and 100%) and dried using tetramethylsilane (Sigma-Aldrich, St. Louis, MO, USA). Finally, the samples were mounted onto stubs and sputter-coated with platinum. Observations were performed with a Hitachi S–4300 scanning electron microscope (Hitachi, Tokyo, Japan). The previous studies were referred to for instructions on morphological comparisons [31–41]. Strains that did not match those in the published literature were treated as unidentified species.

### DNA extraction, PCR and sequencing

For DNA extraction, the cultured strain (100 μl) was harvested by centrifugation at 14,000 × g for 1 min and the cell pellet was resuspended in 1 ml of sterilized STE (sodium chloride-Tris-EDTA, pH 7.8) buffer solution. Two cycles of freezing (–80°C) and thawing (95°C) were followed by vigorous vortexing with sterilized silica/zirconium beads to break the cells. To remove cell debris, the lysate was centrifuged at 8,000 × g for 1 min. The supernatant was dispensed into a clean tube and used as template DNA for PCR.

PCR amplification was performed using two primer sets: Diatom9F (5′–TGTGGGAGAGGGGAAATCAAG–3′) [42] and EukB-R (5′–TGATCCTTCTGCAGGTTCACCAC–3′) [15] for 18S rDNA, and DPrbcL1 (5′–AAGGAGAAATHAATGTCT–3′) and DPrbcL7 (5′–AARCAACCTTGTGTAAGTCTC–3′) for the *rbcL* gene [43]. These primers produced PCR products of approximately 1,600 bp and 1,550 bp, respectively. PCR was performed in a total volume of 30 μl, containing 1.0 μl of template DNA, 3 μl of 10 × Ex Taq buffer, 2.4 μl of dNTPs (10 mM), 0.5 μl of each primer (10 μM), and 0.2 μl of TaKaRa Ex Taq polymerase (5 U μl^−1^; Takara, Otsu, Japan). PCR was conducted using the following conditions: PCR of 18S rRNA was conducted with initial denaturation at 94°C for 5 min, 34 cycles of main amplification (94°C for 45 sec, 55°C for 55 sec, 72°C for 2 min), and final extension at 72°C for 10 min. PCR of *rbcL* was conducted with initial denaturation at 94°C for 3 min, 35 cycles of main amplification (94°C for 1 min, 55°C for 1 min, 72°C for 1.5 min), and final extension at 72°C for 10 min. PCR products were purified using the Accuprep PCR Purification Kit (Bioneer, Daejeon, South Korea) and sent for commercial sequencing at Macrogen (Seoul, South Korea). The electrophenogram outputs for each product were edited and assembled using the ChromasPro v.1.45 program (www.technelysium.com.au/chromas.html) and Vector NTI Advance 11 (Invitrogen Corp., Carlsbad, CA, USA). The sequences obtained in this study were deposited in GenBank and the accession numbers of the sequences are shown in Table 1.

**Table 1:** Strains of the benthic diatoms isolated in this study, information on their collection, and accession numbers of 18S rRNA and *rbcL* gene sequences. Species names were determined by morphological analyses.

Species name by morphological characteristics	Strain designation	Collection information (Location / Date)	Accession number	
			18S	*rbcL*
*Bacillaria paxillifer* (O.F. Müller) T. Marsson	EW234	Eulwang-ri, Incheon, Korea / 20 Apr 2012	KY320376	KY320315
*Unidentified Bacillaria* sp.1	SH349	Sihwa, Siheung, Korea / 8 Mar 2013	KY320377	KY320316
*Cylindrotheca closterium* (Ehrenberg) Reimann & J.C. Lewin	TA256	Geunso Bay, Taean, Korea / 11 Apr 2011	KY320373	KY320312
*Cylindrotheca gracilis* (Brébisson *ex* Kützing) Grunow	TA46	Geunso Bay, Taean, Korea / 21 Jan 2011	KY320374	KY320313
*Unidentified Cylindrotheca* sp.1	TA198	Geunso Bay, Taean, Korea / 23 Mar 2011	KY320375	KY320314
*Nitzschia aequorea* Hustedt	Dilu38	Geunso Bay, Taean, Korea / 22 Mar 2012	KY320391	KY320330
*Nitzschia bergii* A. Cleve	TA139	Geunso Bay, Taean, Korea / 23 Mar 2011	KY320379	KY320318
*Nitzschia dissipata* (Kützing) Rabenhorst	TA44 TA192	Geunso Bay, Taean, Korea / 21 Jan 2011 Geunso Bay, Taean, Korea / 23 Mar 2011	KY320393 KY320394	KY320332 KY320333
*Nitzschia dubia* W. Smith	TA37	Geunso Bay, Taean, Korea / 21 Jan 2011	KY320381	KY320321
*Nitzschia dubiiformis* Hustedt	SH366	Sihwa, Siheung, Korea / 8 Mar 2013	KY320382	KY320320
*Nitzschia liebetruthii* Rabenhorst	TA353	Geunso Bay, Taean, Korea / 23 Feb 2012	KY320378	KY320331
*Nitzschia ligowskii* Witkowski, Lange-Bertalot, Kociolek & Brzezinska	TA426	Geunso Bay, Taean, Korea / 5 Dec 2013	KY320392	KY320317
*Nitzschia paleaeformis* Hustedt	TA394	Geunso Bay, Taean, Korea / 22 Mar 2012	KY320383	KY320322
*Nitzschia* cf. *paleacea*	TA406	Geunso Bay, Taean, Korea / 22 Jan 2014	KY320380	KY320319
*Nitzschia pellucida* Grunow	EW229	Eulwang-ri, Incheon, Korea / 20 Apr 2012	KY320389	KY320328
*Nitzschia pusilla* Grunow	TA-45 TA420	Geunso Bay, Taean, Korea / 17 Apr 2014 Geunso Bay, Taean, Korea / 5 Dec 2013	KY320384 KY320390	KY320323 KY320329
*Nitzschia sigma* (Kützing) W. Smith	TA341 TA377	Geunso Bay, Taean, Korea / 23 Feb 2012 Geunso Bay, Taean, Korea / 22 Mar 2012	KY320395 KY320385	KY320337 KY320324
*Nitzschia sigmaformis* Hustedt	TA311	Geunso Bay, Taean, Korea / 27 Jan 2012	KY320386	KY320325
*Unidentified Nitzschia* sp.1	Dilu16	Geunso Bay, Taean, Korea / 22 Mar 2012	KY320387	KY320326
*Unidentified Nitzschia* sp.2	TA61	Geunso Bay, Taean, Korea / 21 Jan 2011	KY320388	KY320327
*Unidentified Nitzschia* sp.4	TA409	Geunso Bay, Taean, Korea / 22 Jan 2014	KY320396	KY320338
*Tryblionella apiculata* Gregory	TA-85	Geunso Bay, Taean, Korea / 17 Apr 2014	KY320397	KY320334
*Berkeleya fennica* Juhlin-Dannfelt	TA424	Geunso Bay, Taean, Korea / 5 Dec 2013	KY320346	KY320285
*Berkeleya rutilans* (Trentepohl *ex* Roth) Grunow	TA440	Geunso Bay, Taean, Korea / 5 Dec 2013	KY320345	KY320284
*Parlibellus delognei* (Van Heurck) E.J. Cox	TA387	Geunso Bay, Taean, Korea / 22 Mar 2012	KY320352	KY320291
*Haslea nipkowii* (Meister) M. Poulin & G. Massé	SH381	Sihwa, Siheung, Korea / 8 Mar 2013	KY320351	KY320290
*Haslea pseudostrearia* Massé, Rincé & E.J. Cox	TA280	Geunso Bay, Taean, Korea / 11 Apr 2011	KY320350	KY320289
*Navicula agatkae* Witkowski	TA291	Geunso Bay, Taean, Korea / 11 Apr 2011	KY320353	KY320292
*Navicula flagellifera* Hustedt	TA105	Geunso Bay, Taean, Korea / 10 Feb 2011	KY320357	KY320296
*Navicula gregaria* Donkin	TA289	Geunso Bay, Taean, Korea / 11 Apr 2011	KY320358	KY320297
*Navicula incertata* Lange-Bertalot	TA414	Geunso Bay, Taean, Korea / 5 Dec 2013	KY320359	KY320298
*Navicula perminuta* Grunow	TA413 TA441	Geunso Bay, Taean, Korea / 5 Dec 2013 Geunso Bay, Taean, Korea / 5 Dec 2013	KY320360 KY320361	KY320299 KY320300
*Navicula ramosissima* (C. Agardh) Cleve	TA316 TA439	Geunso Bay, Taean, Korea / 27 Jan 2012 Geunso Bay, Taean, Korea / 5 Dec 2013	KY320362 KY320363	KY320301 KY320302
*Navicula salinarum* Grunow	TA402	Geunso Bay, Taean, Korea / 22 Jan 2014	KY320364	KY320303
*Navicula salinarum* var. *minima* R. Kolbe	TA416	Geunso Bay, Taean, Korea / 22 Jan 2014	KY320365	KY320304
*Navicula* cf. *salinarum*	TA407	Geunso Bay, Taean, Korea / 22 Jan 2014	KY320354	KY320293
*Navicula salinicola* Hustedt	TA204	Geunso Bay, Taean, Korea / 23 Mar 2011	KY320366	KY320305
*Navicula trivialis* Lange-Bertalot	TA83	Geunso Bay, Taean, Korea / 21 Jan 2011	KY320372	KY320311
*Unidentified Navicula* sp. 1	TA298	Geunso Bay, Taean, Korea / 11 Apr 2011	KY320367	KY320306
*Unidentified Navicula* sp. 2	TA64	Geunso Bay, Taean, Korea / 21 Jan 2011	KY320368	KY320307
*Unidentified Navicula* sp. 3	EW220	Eulwang-ri, Incheon, Korea / 20 Apr 2012	KY320370	KY320309
*Unidentified Navicula* sp. 4	TA323	Geunso Bay, Taean, Korea / 27 Jan 2012	KY320369	KY320308
*Unidentified Navicula* sp. 5	TU3	Geunso Bay, Taean, Korea / 5 Dec 2013	KY320371	KY320310
*Unidentified Navicula* sp. 6	TA308 TA446	Geunso Bay, Taean, Korea / 27 Jan 2012 Geunso Bay, Taean, Korea / 5 Dec 2013	KY320355 KY320356	KY320294 KY320295
*Unidentified Seminavis* sp.	TA305	Geunso Bay, Taean, Korea / 27 Jan 2012	KY320398	KY320335
*Gyrosigma limosum* Sterrenburg & Underwood	TA152 TA400	Geunso Bay, Taean, Korea / 23 Mar 2011 Geunso Bay, Taean, Korea / 22 Jan 2014	KY320347 KY320348	KY320347 KY320348
*Unidentified Pleurosigma* sp.	TA34	Geunso Bay, Taean, Korea / 21 Jan 2011	KY320349	KY320288
*Entomoneis paludosa*	TA208 TA263	Geunso Bay, Taean, Korea / 23 Mar 2011 Geunso Bay, Taean, Korea / 11 Apr 2011	KY320339 KY320340	KY320278 KY320279
*Unidentified Entomoneis* sp. 1	TA410	Geunso Bay, Taean, Korea / 22 Jan 2014	KY320341	KY320280
*Unidentified Entomoneis* sp. 2	TA350 SH373	Geunso Bay, Taean, Korea / 23 Feb 2012 Sihwa, Siheung, Korea / 8 Mar 2013	KY320343 KY320342	KY320282 KY320281
*Unidentified Entomoneis* sp. 3	EW239	Eulwang-ri, Incheon, Korea / 20 Apr 2012	KY320344	KY320283
*Petrodictyon gemma* (Ehrenberg) D.G. Mann	TA201	Geunso Bay, Taean, Korea / 23 Mar 2011	KY320399	KY320336

### Sequence alignment and phylogenetic analyses

For phylogenetic analysis, 18S rRNA and *rbcL* sequences from diatoms were retrieved in GenBank (www.ncbi.nlm.nih.gov). After excluding uncultured and environmental clone sequences, 1,853 sequences of the 18S rRNA gene and 1,473 sequences of the *rbcL* gene were aligned with the sequences obtained in present study using the ARB program [44] and corrected manually. Two Ochrophyta species (*Nannochloropsis salina* D.J. Hibberd and *Ochromonas danica* E.G. Pringsheim) were used as an outgroup. Neighbor–joining (NJ) and maximum–parsimony (MP) trees were constructed using MEGA 5.2 [45]. Maximum–likelihood (ML) trees were constructed using Randomized Axelerated Maximum Likelihood (RAxML) v.8.2.1 [46]. We used the “–f a” option for rapid bootstrap analysis and the best likelihood tree search using “–# 100” with default settings, namely, “–m GTRGAMMA” for the substitution model with rate heterogeneity, “–i” for the automatically optimized SPR rearrangement for heuristic search, and “–c” for 25 distinct rate categories. The robustness of each clade was assessed by further bootstrap analyses (1,000 replications) under the NJ and MP criteria using MEGA v.5.2 [45].

## Results

### Morphological observations

**Fig 2:**
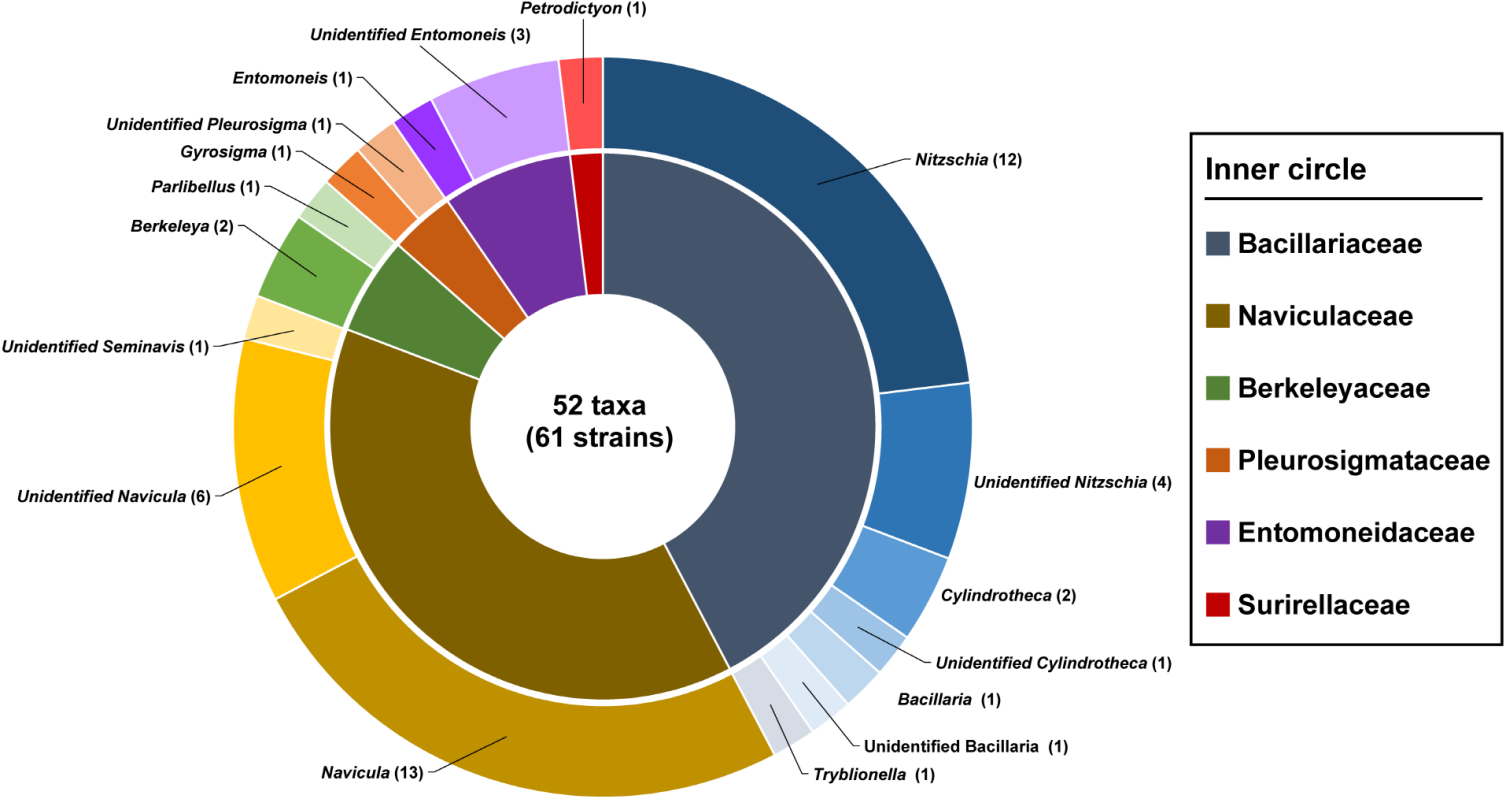
Pie chart showing morphological affiliations of the strains isolated in this study.

**Fig 3:**
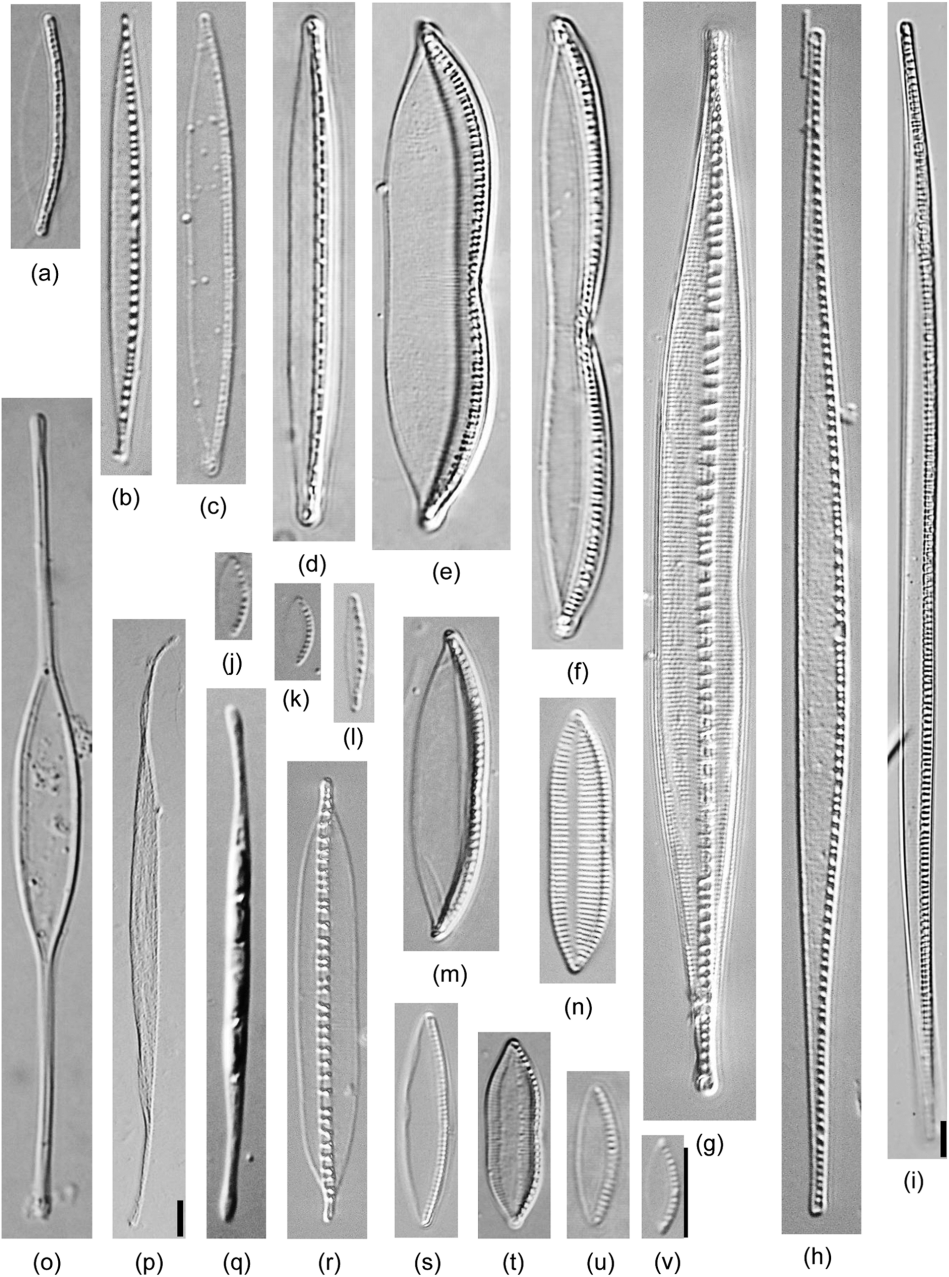
Light micrograph of diatoms isolated in this study belonging to *Cylindrotheca*, *Nitzschia*, and *Tryblionella*. (a) *Nitzschia paleacea* TA406. (b) *N*. *paleaeformis* TA394. (c) *N*. *dubiiformis* SH366. (d) *N*. *dissipata* TA44. (e) *N*. *dubia* TA37. (f) *N*. *pellucida* EW229. (g) *Bacillaria* sp.1 SH349. (h) *N*. *sigmaformis* TA311. (i) *Nitzschia sigma* TA341 (400x). (j) *Nitzschia* sp.1 Dillu16. (k) *Nitzschia* sp.2 TA61. (l) *N*. *liebetruthii* TA353. (m) *Nitzschia* sp.4 TA409. (n) *Tryblionella apiculate* TA-85. (o) *Cylindrotheca closterium* TA256. (p) *C*. *gracilis* TA46 (400x). (q) *Cylindrotheca* sp.1 TA198. (r) *Bacillaria paxillifer* EW234. (s) *Nitzschia bergii* TA139. (t) *N*. *ligowskii* TA426. (u) *N*. *pusilla* TA420. (v) *N*. *aequorea* Dillu38. Scale bar = 10 μm. Note that scale bars of 9 and 16 are inside of the picture.

**Fig 4:**
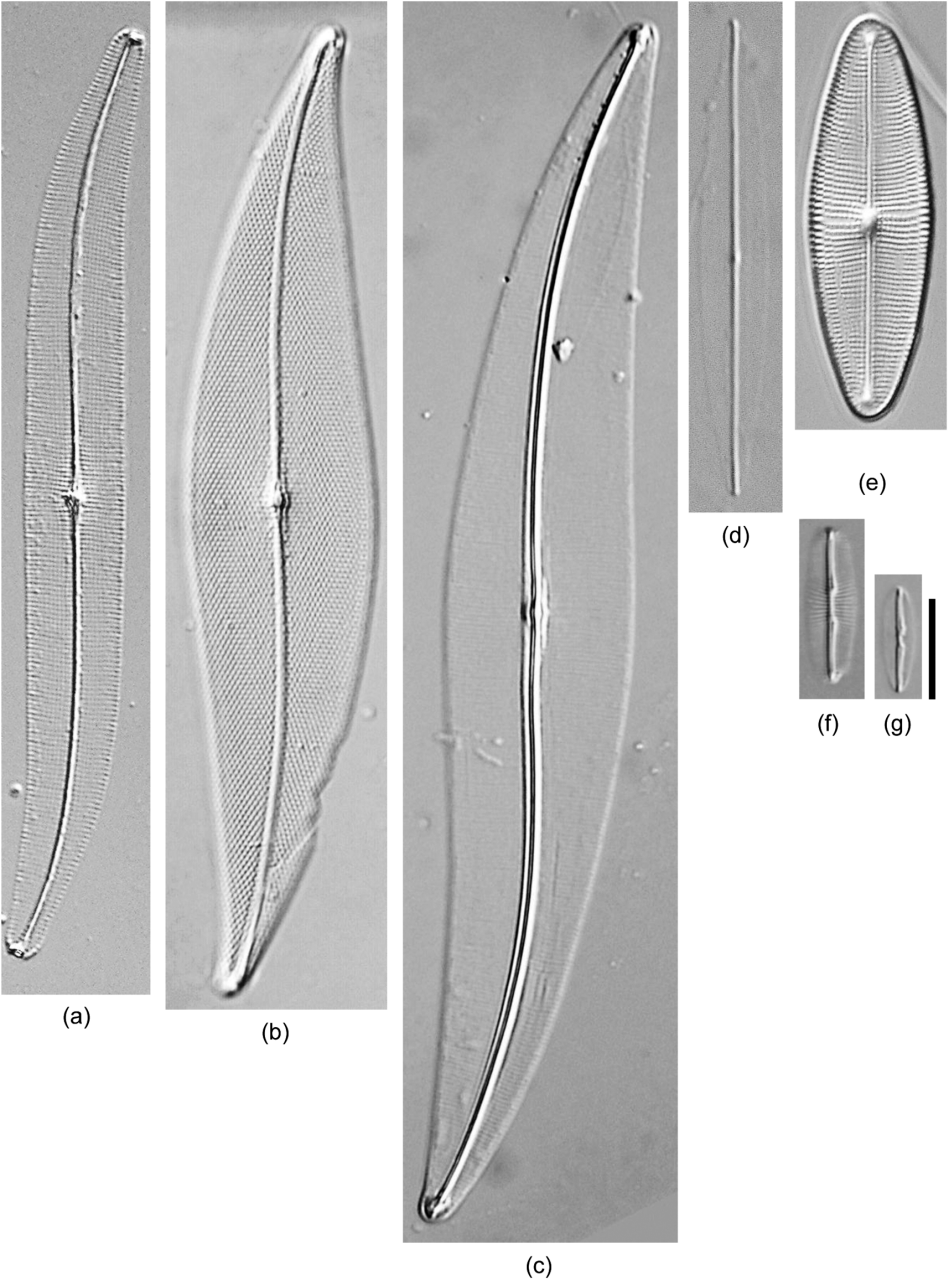
Light micrograph of diatoms isolated in this study belonging to *Berkeleya*, *Gyrosigma*, *Haslea*, *Parlibellus*, and *Pleurosigma*. (a) *Gyrosigma limosum* TA152. (b) *Pleurosigma* sp.1 TA34. (c) *Haslea pseudostrearia TA280*. (d) *H*. *nipkowii* SH381. (e) *Parlibellus delognei* TA387. (f) *Berkeleya rutilans* TA440. (g) *B*. *fennica* TA424. Scale bar = 10 μm.

**Fig 5:**
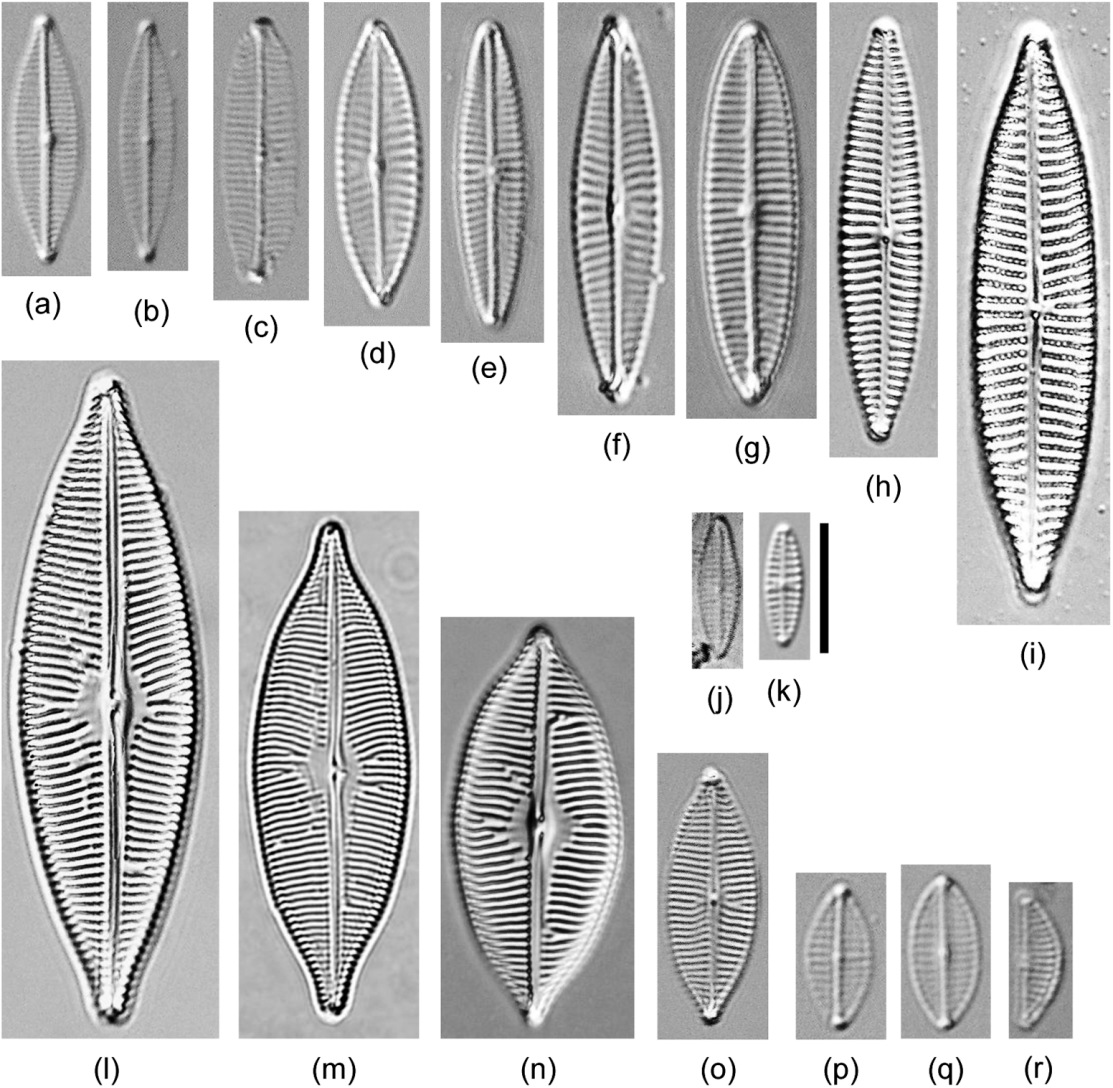
Light micrograph of diatoms isolated in this study belonging to *Navicula* and *Seminavis*. (a) *Navicula gregaria* TA289. (b) *N*. *agatkae* TA291. (c) *Navicula incertata* TA414. (d) *Navicula* sp.1 TA298. (e) *Navicula* sp.5 TU3. (f) *Navicula* sp.3 EW220. (g) *N*. *ramosissima* TA316. (h) *N*. *flagellifera* TA105. (i) *Navicula* sp.2 TA64. (j) *N*. *salinicola* TA204. (k) *N*. *perminuta* TA441. (l) *N*. *trivialis* TA83. (m) *N*. *salinarum* TA402. (n) *N*. cf. *salinarum* TA407. (o) *N*. *salinarum* var. *minima* TA416. (p) *Navicula* sp.4 TA323. (q) *Navicula* sp.6 TA446. (r) *Seminavis* sp.1 TA305. Scale bar = 10 μm.

**Fig 6:**
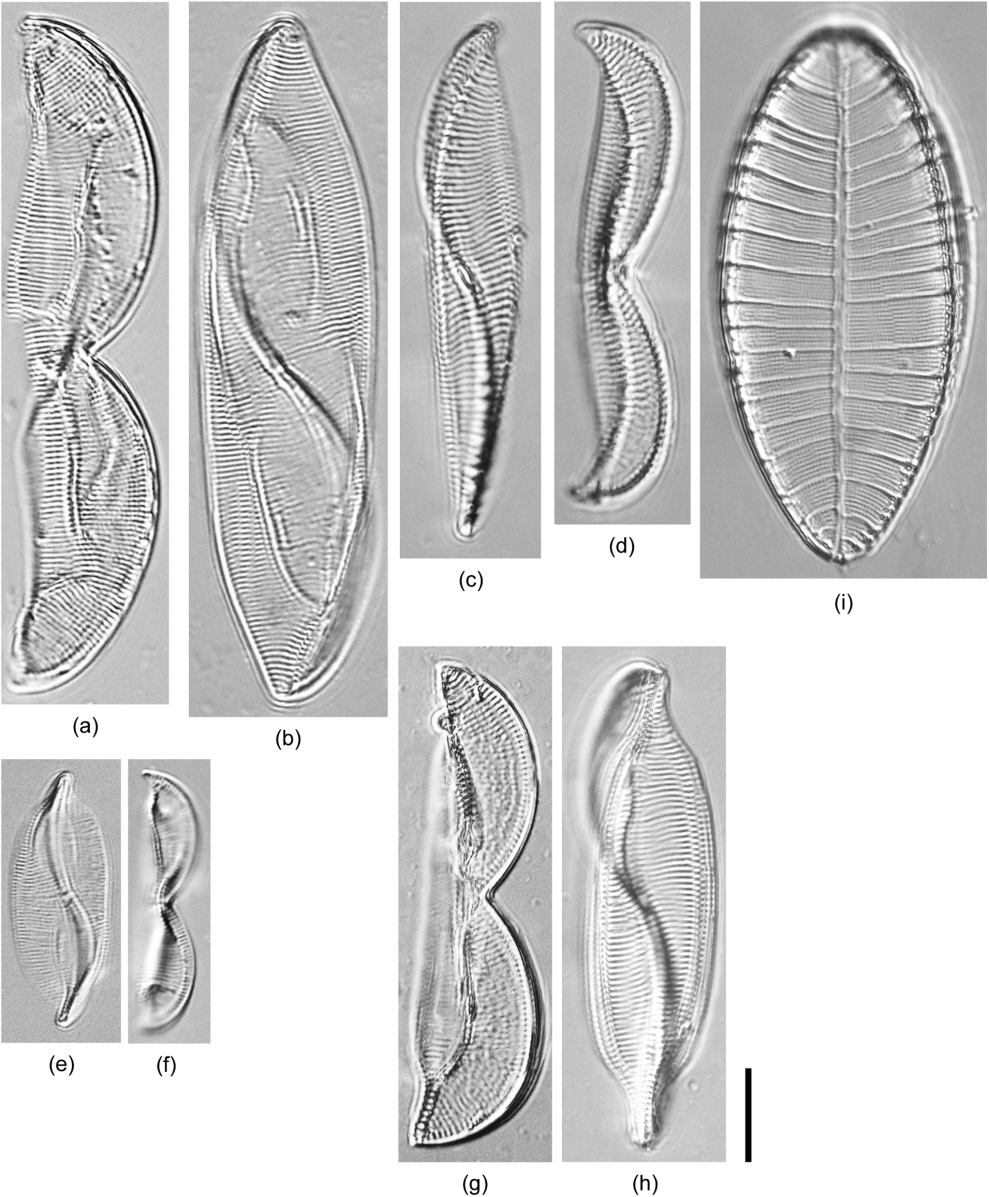
Light micrograph of diatoms isolated in this study belonging to *Entomoneis* and *Petodictyon*. (a, b) *Entomoneis paludosa* TA208. (c) *Entomoneis* sp.2 SH373. (d, e) *Entomoneis* sp.3 EW239. (f, g) *Entomoneis* sp.1 TA410. (h) *Petrodictyon gemma* TA201. Scale bar = 10 μm.

The 61 diatom isolates were identified by morphometric characteristics using light and scanning electron microscopy and their detailed information is shown in Table 2. All strains were raphid diatoms and classified into 3 orders, 6 families, 13 genera, and 52 taxa (36 known and 16 unknown taxa; Fig 2). Forty-two strains could be morphologically identified to the species level (Table 2). Most isolates belonged to Bacillariaceae (25 isolates under 4 genera, 22 taxa) or Naviculaceae (23 isolates under 3 genera, 20 taxa), and the rest belonged to 4 classes, namely, Berkeleyaceae (3 isolates under 2 genera, 3 taxa), Entomoneidaceae (6 isolates under *Entomoneis*, 4 taxa), Pleurosigmataceae (3 isolates under 2 genera, 2 taxa), and Surirellaceae (1 isolate under 1 taxon). *Navicula* (17 taxa) and *Nitzschia* (16 taxa) were abundant in new isolates, followed by *Entomoneis* (4 taxa), *Cylindrotheca* (3 taxa), *Bacillaria* (2 taxa), *Berkeleya* (2 taxa), and *Halsea* (2 taxa). Based on the morphological observations, 42 strains (69%) were identified as 35 known taxa; however, 19 strains (31%) remained as 16 unidentified taxa, namely, 6 *Navicula*, 3 *Nitzschia*, 3 *Entomoneis*, and 1 each for *Bacillaria*, *Cylindrotheca*, *Pleurosigma* and *Seminavis*. The recognized identities and observed morphometric characteristics of the strains are summarized in Table 2; light micrographs of diatoms of the various taxa are shown in Figs 3–6.

**Table 2:** Morphometric data and classification based on the morphology of diatom strains isolated in this study. Species name and sequence identity of the closest relative found in GenBank using BLASTn.

Species name	Strain no.	Morphometrics							Ref.	BLASTn			
		L^1^ (μm)	W^2^ (μm)	Striae in 10 μm			L^6^ /10 μm	F^7^ /10 μm		18S rDNA		*rbcL* gene	
				T^3^	L^4^	O^5^				Species name	Ident (%).	Species name	Ident. (%)
**Bacillariales Hendey**													
**Bacillariaceae Ehrenberg**													
*Bacillaria paxillifer* (O.F. Müller) T. Marsson	EW234	55.7	6.5	23			20	7	[37]	*Bacillaria paxillifer*	99.9	*Bacillaria paxillifer*	94.3
Unidentified *Bacillaria* sp.1	SH349	115.7	10.0	20			24	10		*Bacillaria* cf. *paxillifer*	98.5	*Nitzschia lorenziana* Grunow	94.3
*Cylindrotheca closterium* (Ehrenberg) Reimann & J.C. Lewin	TA256	159.4	3.8					15	[34]	*Cylindrotheca closterium*	99.6	*Cylindrotheca* sp.	95.6
*Cylindrotheca gracilis* (Brébisson *ex* Kützing) Grunow	TA46	156.3	4.0					20	[37]	*Cylindrotheca closterium*	98.7	*Cylindrotheca closterium*	95.1
Unidentified *Cylindrotheca* sp.1	TA198	45.6	2.8					20		*Cylindrotheca fusiformis* Reimann & J.C. Lewin	98.7	*Cylindrotheca* sp.	95.0
*Nitzschia aequorea* Hustedt	Dilu38	7.4	3.3	55				22	[34]	*Nitzschia communis* Rabenhorst	99.6	*Nitzschia capitellata* Hustedt	95.1
*Nitzschia bergii* A. Cleve	TA139	25.7	5.3	40			22	16	[37]	*Nitzschia bizertensis* B. Smida, N. Lundholm, A.S. Hlaili & H.H. Mabrouk	99.6	*Nitzschia palea* (Kützing) W. Smith	95.3
*Nitzschia dissipata* (Kützing) Rabenhorst	TA44 TA192	37.8 41.4	5.8 6.1	48				6	[34]	*Nitzschia epithemoides* Grunow	97.6 97.0	*Nitzschia sigmoidea* (Nitzsch) W. Smith	95.6 96.5
*Nitzschia dubia* W. Smith	TA37	55.2	12.0	23				10	[37]	*Nitzschia dubiformis* Hustedt	99.8	*Psammodictyon constrictum* (Gregory) D.G. Mann	94.6
*Nitzschia dubiiformis* Hustedt	SH366	48.3	5.0	38				16	[37]	*Nitzschia dubiformis*	98.7	*Nitzschia dubiiformis*	93.4
*Nitzschia liebetruthii* Rabenhorst	TA353	21.1	2.7	21				11	[37]	*Nitzschia ovalis* H.J. Arnott	96.7	*Bacillaria paxillifer*	94.5
*Nitzschia ligowskii* Witkowski, Lange-Bertalot, Kociolek & Brzezinska	TA426	22.0	7.6	26				11	[38]	*Nitzschia apiculata* (Gregory) Grunow	98.9	*Tryblionella apiculata*	95.3
*Nitzschia paleaeformis* Hustedt	TA394	50.0	4.5	36				9	[34]	*Nitzschia* sp.	98.8	*Tryblionella apiculata*	94.1
*Nitzschia* cf. *paleacea*	TA406	19.1	5.0	48				8	[34]	*Bacillaria* cf. *paxillifer*	98.9	*Tryblionella apiculata*	94.1
*Nitzschia pellucida* Grunow	EW229	73.7	7.5	33				14	[37]	*Nitzschia dubiformis*	99.2	*Psammodictyon constrictum*	92.9
*Nitzschia pusilla* Grunow	TA-45 TA420	20.0 45.5	4.4 4.9	51 53				18 20	[34]	*Nitzschia thermalis* (Ehrenberg) Auerswald	99.3 99.3	*Nitzschia capitellata*	95.3 95.6
*Nitzschia sigma* (Kützing) W. Smith	TA341 TA377	277.1 303.3	8.2 8.4	28 30				6	[37]	*Nitzschia bizertensis*	96.0 96.1	*Nitzschia capitellata*	94.8 94.9
*Nitzschia sigmaformis* Hustedt	TA311	84.8	5.2	27				10	[37]	*Nitzschia palea*	97.5	*Nitzschia filiformis* (W. Smith) Hustedt	91.2
Unidentified *Nitzschia* sp.1	Dilu16	11.3	3.7	52				18		*Nitzschia thermalis*	99.2	*Nitzschia capitellata*	95.2
Unidentified *Nitzschia* sp.2	TA61	9.8	3.0	56				20		*Nitzschia thermalis*	99.5	*Nitzschia capitellata*	95.0
Unidentified *Nitzschia* sp.4	TA409	26.9	7.8	40				12		*Nitzschia dubiiformis*	99.7	*Psammodictyon constrictum*	94.3
*Tryblionella apiculata* Gregory	TA-85	30.8	6.7	16					[34]	*Nitzschia apiculata*	99.6	*Tryblionella apiculata*	96.5
**Naviculales Bessey**													
**Berkeleyaceae D.G.Mann**													
*Berkeleya fennica* Juhlin-Dannfelt	TA424	11.7	3.9	36^9^					[37]	*Berkeleya rutilans*	99.4	*Berkeleya rutilans*	94.7
*Berkeleya rutilans* (Trentepohl *ex* Roth) Grunow	TA440	15.9	4.1	28^9^					[37]	*Berkeleya rutilans*	99.8	*Berkeleya rutilans*	99.5
*Parlibellus delognei* (Van Heurck) E.J. Cox	TA387	36.7	12.7	19					[32]	*Prestauroneis integra* (W. Smith) K. Bruder	98.92	*Craticula cuspidata* (Kützing) D.G. Mann	94.2
**Naviculaceae Kützing**													
*Haslea nipkowii* (Meister) M. Poulin & G. Massé	SH381	130.5	17.8	26	28				[41]	*Haslea nipkowii*	99.7	*Haslea* sp.	95.9
*Haslea pseudostrearia* Massé, Rincé & E.J. Cox	TA280	48.4	6.0	41	35				[39]	*Haslea pseudostrearia*	100.0	*Haslea pseudostrearia*	96.7
*Navicula agatkae* Witkowski	TA291	18.7	4.7	18			15		[37]	*Navicula gregaria*	99.5	*Navicula* sp. S0020	96.0
*Navicula flagellifera* Hustedt	TA105	33.6	6.4	14			40		[37]	*Navicula* sp.	99.9	*Navicula* sp. S0020	99.2
*Navicula gregaria* Donkin	TA289	25.5	5.2	18			29		[33]	*Navicula gregaria*	99.9	*Seminavis* cf. *robusta*	95.4
*Navicula incertata* Lange-Bertalot	TA414	19.2	3.5	16			48		[33]	*Navicula tripunctata* (O.F. Müller) Bory de Saint-Vincent	99.6	*Navicula* sp. S0020	95.6
*Navicula perminuta* Grunow	TA413 TA441	4.3 5.6	2.0 2.0	26			40		[37]	*Navicula perminuta*	100.0	*Seminavis* cf. *robusta*	94.0 93.9
*Navicula ramosissima* (C. Agardh) Cleve	TA316 TA439	25.1 30.8	5.7 6.9	12 15			38 40		[37]	*Navicula arenaria*	99.8 99.5	*Navicula ramosissima*	97.7 97.3
*Navicula salinarum* Grunow	TA402	37.0	11.7	15			33		[37]	*Navicula phyllepta* Kützing	99.5	*Navicula cryptocephala* Kützing	96.0
*Navicula salinarum* var. *minima* R. Kolbe	TA416	20.3	6.9	18			42		[37]	*Navicula phyllepta*	99.6	*Navicula cryptocephala*	95.4
*Navicula* cf. *salinarum*	TA407	36.3	13.8	14			31			*Navicula phyllepta*	99.5	*Navicula cryptocephala*	95.6
*Navicula salinicola* Hustedt	TA204	12.3	3.5	20			40		[37]	*Navicula lanceolate* (C. Agardh) Kützing	99.5	*Navicula* sp. S0020	95.8
*Navicula trivialis* Lange-Bertalot	TA83	49.0	12.5	14			30		[33]	*Navicula phyllepta*	99.5	*Navicula cryptocephala*	95.2
Unidentified *Navicula* sp. 1	TA298	24.0	6.3	12						*Navicula ramosissima*	99.6	*Navicula* sp. S0020	95.5
Unidentified *Navicula* sp. 2	TA64	36.8	10.4	9						*Navicula veneta* Kützing	98.6	*Navicula* sp. S0020	95.7
Unidentified *Navicula* sp. 3	EW220	29.5	7.1	12			20			*Navicula lanceolata *	99.6	*Navicula* sp. S0020	95.6
Unidentified *Navicula* sp. 4	TA323	11.6	5.0	18			36			*Navicula* sp.	99.6	*Navicula ramosissima*	96.9
Unidentified *Navicula* sp. 5	TU3	10.6	5.0	16			39			*Navicula* sp.	99.9	*Navicula* sp. S0020	99.6
Unidentified *Navicula* sp. 6	TA308 TA446	11.8	5.6	20			45 46			*Navicula arenaria* Donkin	99.7 99.5	*Navicula* sp. S0020	95.7 95.6
Unidentified *Seminavis* sp.	TA305	15.9	6.4	18			45			*Navicula phyllepta*	98.9	*Seminavis cf*. *robusta*	95.6
**Pleurosigmataceae Mereschowsky**													
*Gyrosigma limosum* Sterrenburg & Underwood	TA152 TA400	61.6 96.2	10.7 11.2	22 24	2628				[35]	*Gyrosigma acuminatum* (Kützing) Rabenhorst	99.7	*Gyrosigma acuminatum*	96.9
Unidentified *Pleurosigma* sp.	TA34	91.4	20.0		24	20				*Pleurosigma intermedium* W. Smith	98.7	*Gyrosigma acuminatum*	93.9
Surirellales D.G.Mann													
**Entomoneidaceae Reimer in Patrick & Reimer**													
*Entomoneis paludosa*	TA208 TA263	41.4 68.4	35.9^8^	22 23					[31]	*Entomoneis cf*. *alata*	86.5 86.5	*Surirella sp*. *Haslea crucigera* (W. Smith) Simonsen	96.9 96.7
Unidentified *Entomoneis* sp. 1	TA410	30.2	15.7^8^	25^10^						*Entomoneis ornate* (Bailey) Reimer	95.6	*Haslea crucigera*	96.7
Unidentified *Entomoneis* sp. 2	TA350 SH373	50.3 62.8	29.9^8^ 35.2^8^	15^10^ 16^10^						*Enomoneis cf*. *alata* *Entomoneis sp*.	98.7 98.8	*Haslea crucigera*	97.7 97.7
Unidentified *Entomoneis* sp. 3	EW239	53.2	41.0^8^	16^10^						*Amphiprora alata *(Ehrenberg) Kützing	94.6	*Amphiprora alata*	96.5
**Surirellaceae Kützing**													
*Petrodictyon gemma* (Ehrenberg) D.G. Mann	TA201	55.8	25.8						[24]	*Cylindrotheca closterium*	83.8	*Surirella sp*.	96.9

^1^ L, length;

^2^ W, width;

^3^ T, transverse;

^4^ L, longitudinal;

^5^ O, oblique;

^6^ L, lineolae;

^7^ F, fibulae;

^8^ In girdle view;

^9^ In the middle of frustule;

^10^ Striae composed of doubly-punctate striae

### Molecular-based identification

Both 18S rRNA and *rbcL* genes from 61 culture strains were sequenced successfully. The BLASTn results of each 18S rRNA and *rbcL* sequence are given in Table 2 according to the best matched species and sequence identity. For many strains, the closest relative based on the BLAST search differed from identification based on morphology. The morphological and genetic classification results were consistent for only nine strains with >98.7% identity to their closest relatives based on their 18S rRNA gene sequences (Table 2). Similarly, morphological and genetic identification using the *rbcL* sequences were consistent only in six strains with relatively high sequence identities, ranging from 94.3% to 99.5% (Table 2).

**Fig 7:**
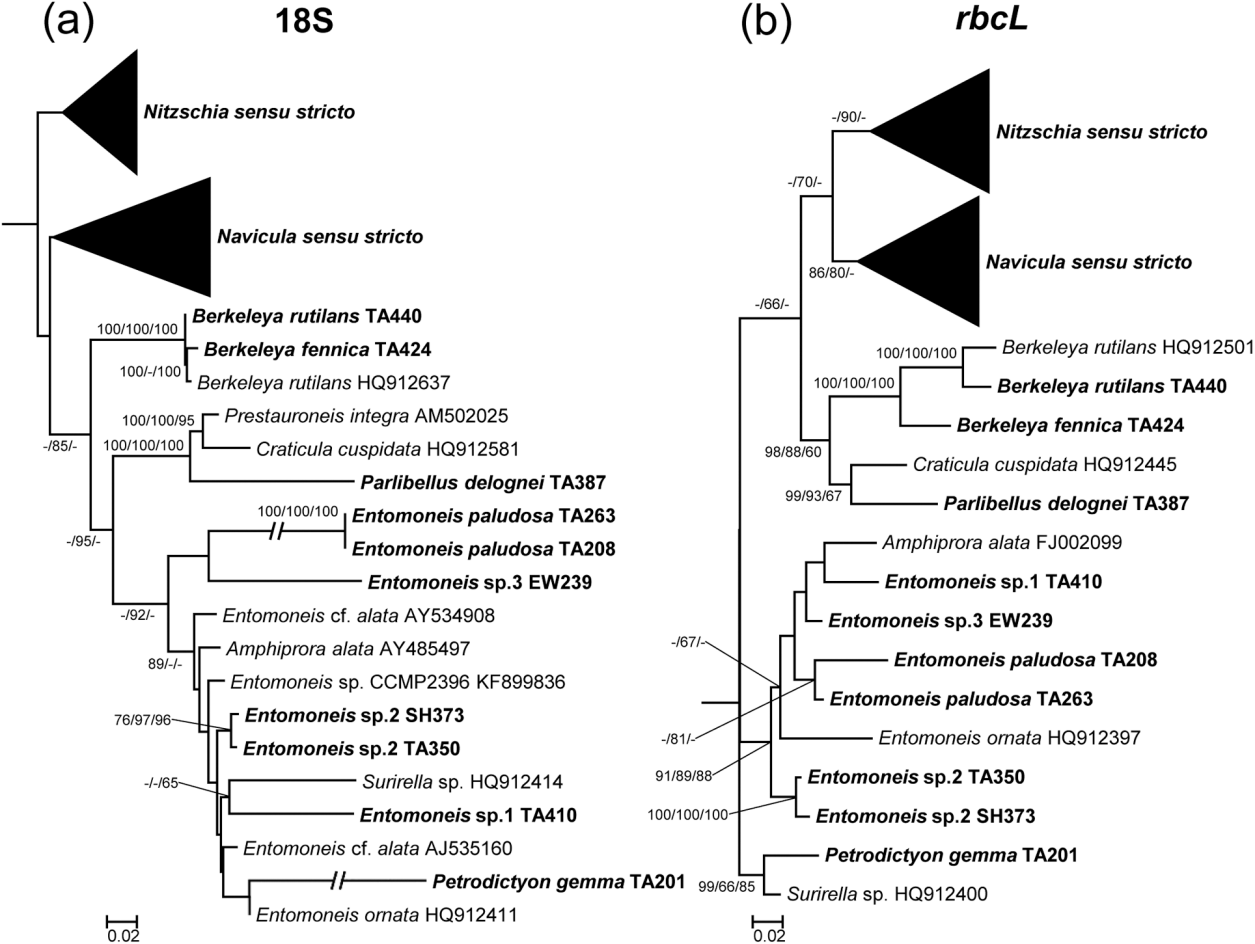
**Phylogenetic trees obtained using 18S rRNA (a) and *rbcL* (b) gene sequences of 61 culture strains.** Bootstrap values obtained by neighbor–joining, maximum–likelihood, and maximum–parsimony methods are shown on the nodes. Expanded tree of *Navicula sensu stricto* and *Nitzschia sensu stricto* are shown in Figs 8 and 9, respectively.

**Fig 8:**
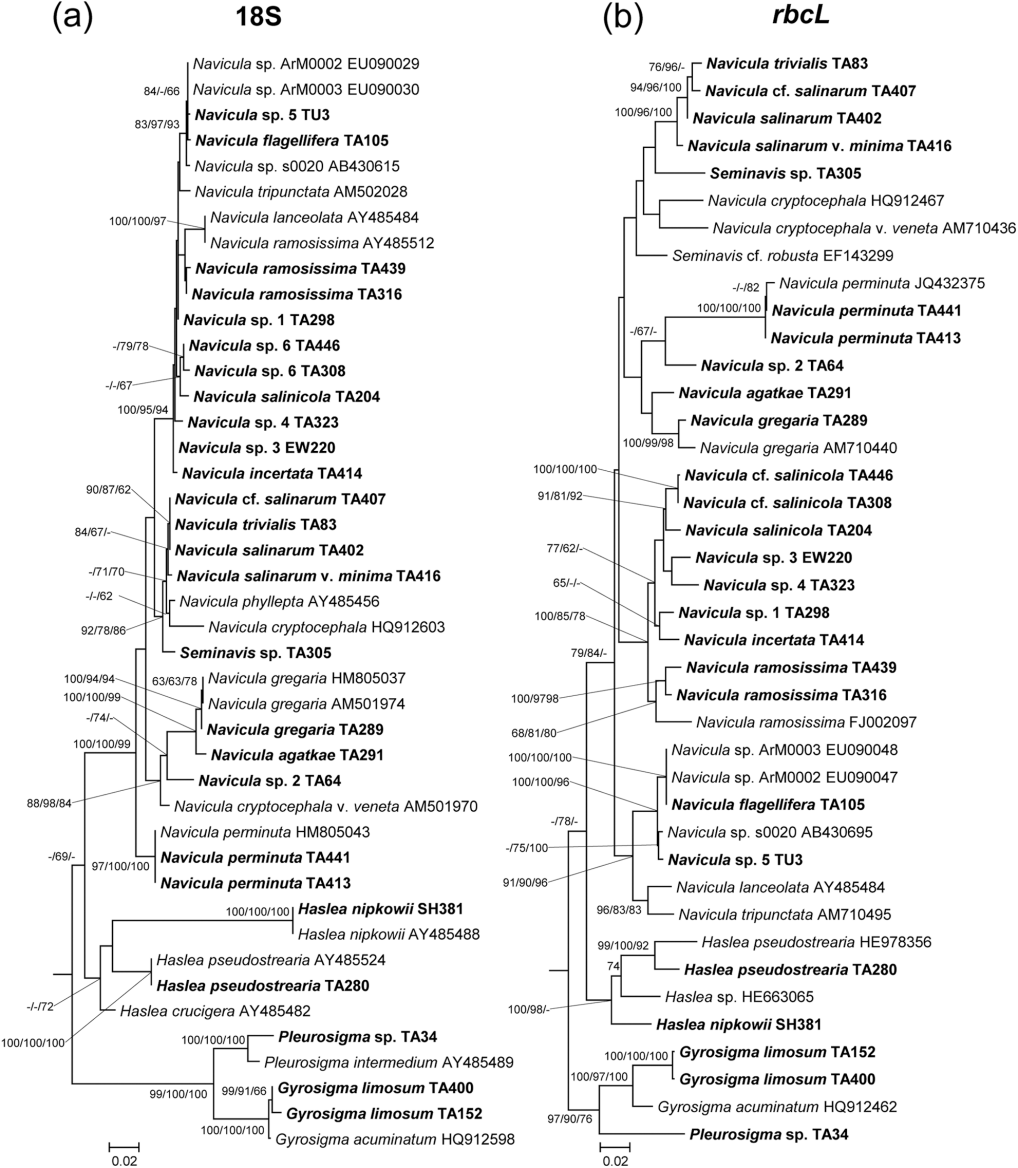
**Phylogenetic tree of *Navicula sensu stricto* obtained using 18S rRNA (a) and *rbcL* (b) gene sequences.** Bootstrap value obtained by neighbor–joining, maximum–likelihood, and maximum–parsimony methods are shown on the nodes.

**Fig 9:**
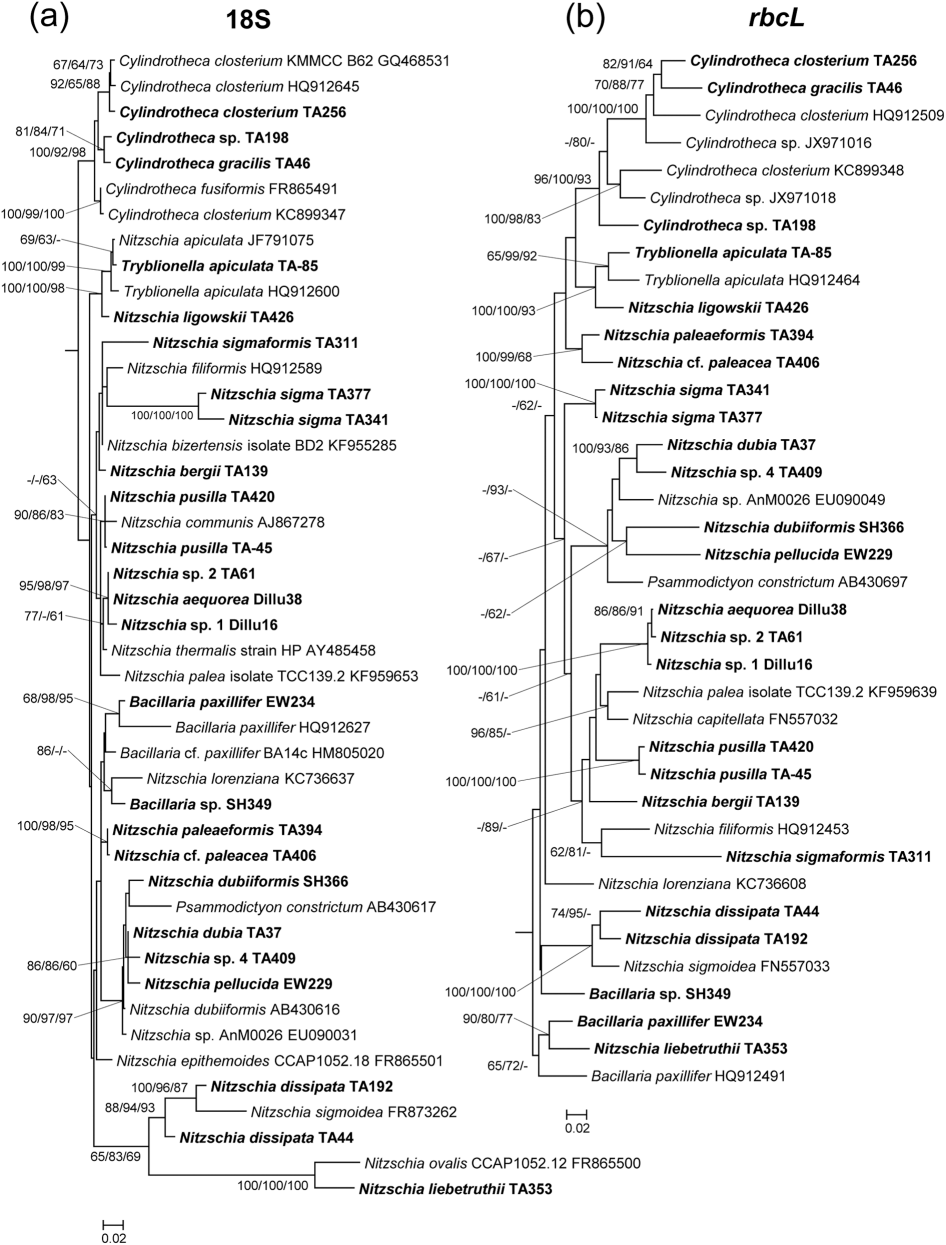
**Phylogenetic tree of *Nitzschia sensu stricto* obtained using 18S rRNA (a) and *rbcL* (b) gene sequences.** Bootstrap value obtained by neighbor–joining, maximum–likelihood, and maximum–parsimony methods are shown on the nodes.

From the phylogenetic trees, phylogenetic relationships among the isolates can be determined (Figs 7–9). In total, 110 sequences of the 18S rRNA gene and 93 sequences of the *rbcL* gene were used for the phylogenetic analysis. In the phylogenetic trees of the *rbcL* gene, most of strains were separated in accordance with their taxonomic positions. In contrast, some strains were not consistent with the morphological classification in the 18S rDNA phylogenies. *Petrodictyon gemma* TA201, belonging to Surirellaceae, clustered with *Entomoneis ornata* strain 14A, belonging to Entomoneidaceae, with a long branch in the ML tree of 18S rDNA (Fig 7). Additionally, two *Entomoneis paludosa* strains, TA208 and TA263, showed another long branch (Fig 7). Unlike the ML tree, however, *P*. *gemma* and the two *E*. *paludosa* strains clustered together with a long branch in the NJ and MP phylogenies. Thus, in the 18S rDNA tree, the phylogenetic positions of these species were unstable. In the Naviculales, despite the fact that the morphological features were similar to those of naviculoids, the tube-dwelling diatoms *Berkeleya* and *Parlibellus* did not cluster in the naviculoid group, but rather in asymmetrical biraphid diatoms with a low bootstrap value in the 18S rDNA phylogenies (Fig 7). In addition, several different species were not clearly differentiated in the 18S rDNA phylogenies, such as *Berkeleya rutilans* TA440 and *Berkeleya fennica* TA424, which had a very low sequence distance (Fig 7, Table 2). A similar low resolution was also found among *Navicula salinarum* TA402, *Navicula trivialis* TA83, and *N*. cf. *trivialis* TA407 (Fig 8).

Using the sequences obtained in this study, we analyzed divergence levels of the 18S rRNA and *rbcL* genes (Table 3). Although the divergence levels of 18S rRNA genes were higher than those of *rbcL* genes in the genus *Entomoneis* due to long branches, the genetic distance of the *rbcL* gene within the genus was, on average, double that of the 18S rRNA gene. Furthermore, the genetic distance of *rbcL* was three times higher than that of 18S rRNA in two dominant benthic genera, *Navicula* and *Nitzschia*.

**Table 3:** Nucleotide sequence distances of the 18s rRNA and *rbcL* genes within a genus according to Jukes and Cantor [47] model.

Order	Genus	No. of strains	Genetic distance	
			18S rDNA	*rbcL*
*Naviculales*	*Navicula*	21	0.015	0.050
	*Haslea*	2	0.037	0.047
	*Berkeleya*	2	0.003	0.049
	*Gyrosigma*	2	0.003	0.021
*Bacillariales*	*Bacillaria*	2	0.022	0.056
	*Nitzschia*	20	0.036	0.078
	*Cylindrotheca*	3	0.010	0.065
*Surirellales*	*Entomoneis*	6	0.074	0.048
Average			0.060	0.089

## Discussion

In this study, we attempted to identify and classify benthic diatoms by the polyphasic approach using both morphological characteristics and molecular markers and suggested that molecular approach using *rbcL* gene could become a better alternative to traditional morphological classification approach. Despite a long history of taxonomic studies on benthic diatoms, overcoming the difficulties associated with identification and classification of diatoms is a major challenge because of their small size and morphological similarities. In the process of identifying the strains obtained in this study, many strains were not morphologically identified at the species level due to these difficulties. Although more strains might be identifiable by a thorough literature review and some may be confirmed to be a new species, it is evident that morphometric classification is a laborious and time-consuming procedure. Some previous studies avoided identification at the species level or dealt only with the community dynamics of benthic diatoms [12, 13]. Therefore, the community structure of diatoms and their distribution in tidal flats have not been clearly elucidated [48]. To reveal easily and quickly the hidden diversity of benthic diatoms, largely attributed to their very small and similar morphologies, the development of molecular barcoding techniques is urgently needed. To enable this, it is necessary to construct a reliable genetic database.

The quality of a database has a direct and absolute influence on the applicability and efficiency of DNA barcoding techniques [49]. Currently, genetic information on most species could not be found in GenBank, indicating that the database is still insufficient, and that molecular taxonomic studies on benthic diatoms are limited. At the time of writing, the numbers of 18S rDNA and *rbcL* gene sequences deposited in GenBank are 4,775 and 3,099, and the number of species are reduced to 811 and 709, respectively. Despite the fact that extant diatoms are estimated to include 30,000–100,000 species [50], there is no genetic information on the majority of such species. Owing to the limited data available in GenBank, the closest relatives of most 18S rDNA sequences did not match the classifications by morphological identification (Table 2). These inconsistencies were more apparent in the case of the *rbcL* gene.

In this study, six groups of diatoms, namely, Bacillariaceae, Naviculaceae, Pleurosigmataceae, Berkeleyaceae, Entomoneidaceae, and Surirellaceae, were clearly distinguished and formed monophyletic groups in the phylogenetic trees of *rbcL* gene. In the 18S rDNA analyses, despite a morphological difference, some diatom sequences showed high similarity (more than 99%) to those of other species. These relatively high sequence similarities might have been due to either misidentification of records deposited in GenBank or low resolution of the 18S rDNA gene [18, 19]. However, a relatively low sequence distance within a genus shows that 18S rDNA is not an appropriate genetic marker to differentiate diatom species clearly, as is seen in the case of lower resolution among species and polyphyletic characteristics of several species (Table 2). For example, *Navicula salinarum* TA402, *N*. cf. *salinarum* TA407, and *N*. *trivialis* TA83 are similar but morphologically different species. *N*. *trivialis* TA83 has subrostrate apices and a central area that is bound by mostly shortened striae, whereas *N*. *salinarum* TA402 has rostrate apices and a central area that is formed by alternating long and short striae [31, 33]. However, the 18S rDNA sequences of these species are almost identical, and therefore cannot be clearly distinguished from each other (Fig 8). Similarly, *Berkeleya fennica*, which can be distinguished by its smaller and denser striae (over 30/10 μm) from *B*. *rutilans* [40], were not clearly differentiated from *B*. *rutilans* in the 18S rDNA phylogenetic tree. In addition, the Surirelloid diatom *Petrodictyon gemma* was clustered with *Entomoneis* by a long branch in the 18S rDNA phylogeny. This long branch attraction artefact was also found in the 18S rDNA phylogenies of *Haslea nipkowii* and *Neidium affine* [51], indicating that unusually rapid evolutionary events have occurred in the 18S rRNA genes of some benthic diatoms [52]. In this respect, it is apparent that the 18S rRNA gene of some benthic diatoms has undergone unusually rapid evolutionary changes. Thus, although 18S rRNA has been widely used in phylogenetic studies on diatoms and has the largest database compared with other genetic markers [20, 22, 23], it is unsuitable as a marker for the study of diatom biodiversity because of its low resolution [20].

Conversely, the *rbcL* gene varies markedly compared with 18S rDNA [16]. Consistently in this study, the *rbcL* gene showed higher divergence levels than those of the 18S rRNA gene, with a few exceptions in *Entomoneis* and *Haslea*, which were supposed to have undergone rapid evolutionary changes in 18S rDNA (Figs 7 and 8). Furthermore, long branch artefacts were not found among the *rbcL* phylogeny. In addition, the *rbcL* gene, a plastid–encoded gene, is advantageous in its use as a genetic marker because of its high PCR success rate (i.e., ease of amplification), simplicity of alignment, and low susceptibility to interference by heterotrophic contaminants [53]. However, the deficiencies in databases must still be addressed. Hamsher et al. [54] reported that the range of divergence in the *rbcL* gene sequence among species in the genus *Sellaphora* was 0.14–0.73%. Also, Kermarrec et al. [55] suggested 99% and 98% *rbcL* gene sequence identities as the thresholds for species- and genus-level classifications, respectively. However, most strains obtained in this study shared a sequence identity of 97% or less with sequences in the GenBank database. These results indicate that much of the necessary information remains unknown. However, it is still clear that the *rbcL* gene would be more appropriate than 18S rDNA for the molecular taxonomy and phylogenetic analyses of benthic diatoms.

Despite the ecological importance of benthic diatom community, their identification and classification systems still need to be improved. In this study, we showed that a large proportion of diatoms could not be identified by morphological characteristics and that genetic information should be expanded for molecular phylogenetic analyses. Furthermore, *rbcL* gene is suggested as a superior genetic marker to 18S rRNA gene to identify and phylogenetically classify benthic diatoms. The huge number of diatom species estimated in various environments suggests a need for more efforts to construct a reliable database containing polyphasic taxonomic data.

## References

[1] Berger WH, Wefer G. Productivity of the glacial ocean: discussion of the iron hypothesis. Limnol Oceanogr. 1991;36: 1899–1918.

[2] Mann DG. The species concept in diatoms. Phycologia. 1999;38: 437–495.

[3] Dugdale RC, Wilkerson FP, Minas HJ. The role of silicate pump in driving new production. Deep Sea Res I. 1995;42: 697–719.

[4] Gowda G, Gupta T, Rajesh K, Gowda H, Lingadhal C, Ramesh A. Seasonal distribution of phytoplankton in Nethravathi estuary, Mangalore. J Mar Biol Ass India. 2001;43: 31–40

[5] Admiraal W. The ecology of estuarine sediment-inhabiting diatoms. Prog Phycol Res. 1984;3: 269–322.

[6] Underwood GJC, Kromkamp J. Primary production by phytoplankton and microphytobenthos in estuaries. Adv Ecol Res. 1999;29: 93–153.

[7] Haubois AG, Sylvestre F, Guarini JM, Richard P, Blanchard GF. Spatio-temporal structure of the epipelic diatom assemblage from an intertidal mudflat in Marennes-Oléron Bay, France. Estuar Coast Shelf Sci. 2005;64: 385–394.

[8] Hustedt F. Marine littoral diatoms of Beaufort, North Carolina. Duke Univ Mar Stat Bull. 1955;6: 1–67.

[9] Smyth JC. A study of the benthic diatoms of Loch Sween (Argyll). J Ecol. 1955;43: 149–171.

[10] Round FE. Studies on Bottom-Living Algae in Some Lakes of the English Lake District: Part II. The Distribution of Bacillariophyceae on the Sediments. J Ecol. 1957;45: 343–360.

[11] Round FE. Studies on Bottom-Living Algae in Some Lakes of the English Lake District: IV. The Seasonal Cycles of the Bacillariophyceae. J Ecol. 1960;48: 529–547.

[12] Sullivan M, Currin C. Community Structure and Functional Dynamics of Benthic Microalgae in Salt Marshes In: Concepts and Controversies in Tidal Marsh Ecology (Ed. by Weinstein M. & Kreeger D.). Netherlands: Springer; 2000 pp. 81–106.

[13] Brotas V, Plante-cuny MR. The use of HPLC pigment analysis to study microphytobenthos communities. Acta Oecol. 2003;24: S109–S115.

[14] Underwood GJC, Barnett M. What determines species composition in microphytobenthic biofilms? In: Functioning of microphytobenthos in estuaries (Ed. by Kromkamp J.). Amsterdam: Royal Netherlands Academy of Arts and Sciences; 2006 pp. 121–138.

[15] Medlin L, Elwood HJ, Stickel S, Sogin ML. The characterization of enzymatically amplified eukaryotic 16S-like rRNA-coding regions. Gene. 1988;71: 491–499. 3224833 10.1016/0378-1119(88)90066-2

[16] Evans KM, Wortley AH, Mann DG. An assessment of potential diatom "barcode" genes (*cox1*, *rbcL*, 18S and *ITS* rDNA) and their effectiveness in determining relationships in *Sellaphora* (Bacillariophyta). Protist. 2007;158: 349–364. doi: 10.1016/j.protis.2007.04.001 17581782 10.1016/j.protis.2007.04.001

[17] Jahn R, Zetzsche H, Reinhardt R, Gemeinholzer B. Diatoms and DNA barcoding: A pilot study on an environmental sample In: Proceedings of the 1st Central European diatom meeting. Berlin: Freie Universität; 2007 pp. 63–68.

[18] Mann DG, Sato S, Trobajo R, Vanormelingen P, Souffreau C. DNA barcoding for species identification and discovery in diatoms. Cryptogamie Algol. 2010;31: 557–577.

[19] Moniz MBJ, Kaczmarska I. Barcoding of Diatoms: Nuclear Encoded ITS Revisited. Protist. 2010;161: 7–34. doi: 10.1016/j.protis.2009.07.001 19674931 10.1016/j.protis.2009.07.001

[20] Beszteri B, Ács É, Makk J, Kovács G, Márialigeti K, Kiss KT. Phylogeny of six naviculoid diatoms based on 18S rDNA sequences. Int J Syst Evol Microbiol. 2001;51: 1581–1586. doi: 10.1099/00207713-51-4-1581 11491361 10.1099/00207713-51-4-1581

[21] Moniz MBJ, Kaczmarska I. Barcoding diatoms: Is there a good marker?. Mol Ecol Resour. 2009;9: 65–74. doi: 10.1111/j.1755-0998.2009.02633.x 21564966 10.1111/j.1755-0998.2009.02633.x

[22] Jones HM, Simpson GE, Stickle AJ, Mann DG. Life history and systematics of *Petroneis* (Bacillariophyta), with special reference to British waters. Eur J Phycol. 2005;40: 61–87.

[23] Sato S, Kooistra WH, Watanabe T, Matsumoto S, Medlin LK. A new araphid diatom genus *Psammoneis* gen. nov.(Plagiogrammaceae, Bacillariophyta) with three new species based on SSU and LSU rDNA sequence data and morphology. Phycologia. 2008; 47: 510–528.

[24] Pniewski F, Friedl T, Latała A. Identification of diatom isolates from the Gulf of Gdańsk: testing of species identifications using morphology, 18S rDNA sequencing and DNA barcodes of strains from the Culture Collection of Baltic Algae (CCBA). Oceanological and Hydrobiological Studies, 2010;39: 3–20.

[25] Amato A, Kooistra WHCF, Ghiron JHL, Mann DG, Proschold T, Montresor M. Reproductive isolation among sympatric cryptic species in marine diatoms. Protist. 2007;158: 193–207. doi: 10.1016/j.protis.2006.10.001 17145201 10.1016/j.protis.2006.10.001

[26] Trobajo R, Mann DG, Clavero E, Evans KM, Vanormelingen P, Mcgregor RC. The use of partial *cox1*, *rbcL* and LSU rDNA sequences for phylogenetics and species identification within the *Nitzschia palea* species complex (Bacillariophyceae). Eur J Phycol. 2010;45: 413–425.

[27] Ehara M, Inagaki Y, Watanabe KI, Ohama T. Phylogenetic analysis of diatom *coxI* genes and implications of a fluctuating GC content on mitochondrial genetic code evolution. Curr Genet. 2000;37: 29–33. 10672441 10.1007/s002940050004

[28] Delaney JA, Ulrich RM, Paul JH. Detection of the toxic marine diatom *Pseudo*-*nitzschia multiseries* using the RuBisCO small subunit (*rbcS*) gene in two real-time RNA amplification formats. Harmful Algae. 2011;11: 54–64.

[29] Choi DH, Noh JH. Phylogenetic diversity of *Synechococcus* strains isolated from the East China Sea and the East Sea. Fems Microbiol Ecol. 2009;69: 439–448. doi: 10.1111/j.1574-6941.2009.00729.x 19624741 10.1111/j.1574-6941.2009.00729.x

[30] Hendey N. The permanganate method for cleaning freshly gathered diatoms. Microscopy. 1974;32: 423–426.

[31] Patrick R, Reimer CW. The diatoms of the United States: exclusive of Alaska and Hawaii vol. 2 Part 1: Entomoneidaceae, Cymbellaceae, Gomphonemaceae, Epithemiaceae. Pennsylvania: Academy of Natural Sciences of Philadelphia; 1975.

[32] Lobban CS. Marine tube-dwelling diatoms of eastern Canada: descriptions, checklist, and illustrated key. Can J Bot. 1984;62: 778–794.

[33] Krammer K, Lange-Bertalot H. Bacillariophyceae 1. Teil: Naviculaceae In: Süßwasserflora von Mitteleuropa Band 2/1. Heidelberg: Spektrum Akademischer Verlag; 1986.

[34] Krammer K, Lange-Bertalot H. Bacillariophyceae 2. Teil: Bacillariaceae, Epithemiaceae, Surirellaceae In: Süßwasserflora von Mitteleuropa Band 2/2. Heidelberg: Spektrum Akademischer Verlag; 1988.

[35] Sterrenburg FAS, Underwood GJC. Studies on the Genera *Gyrosigma* and *Pleurosigma* (Bacillariophyceae). The Marine "*Gyrosigma spenceri*" Records: *Gyrosigma limosum* Sterrenburg et Underwood nov. sp. Proc Acad Nat Sci Philadelphia. 1997;148: 165–169.

[36] Krammer K, Lange-Bertalot H. Bacillariophyceae English and French translation of the keys In: Süßwasserflora von Mitteleuropa Band 2/5. Heidelberg: Spektrum Akademischer Verlag; 2000.

[37] Witkowski A, Lange-Bertalot H, Metzeltin D. Diatom flora of marine coasts I. Iconographia Diatomologica 7. Königstein: Koeltz Scientific Books; 2000.

[38] Witkowski A, Lange-Bertalot H, Kociolek JP, Ruppel M, Wawrzyniak-Wydrowska B, Bak M, et al Four new species of *Nitzschia* sect. *Tryblionella* (Bacillariophyceae) resembling *N*. *parvula*. Phycologia. 2004;43: 579–595.

[39] Massé G, Rincé Y, Cox E, Allard G, Belt ST, Rowland SJ. *Haslea salstonica* sp. nov. and *Haslea pseudostrearia* sp. nov. (Bacillariophyta), two new epibenthic diatoms from the Kingsbridge estuary, United Kingdom. C R Acad Sci. 2001;324: 617–626. 11476003 10.1016/s0764-4469(01)01330-0

[40] Antoniades D, Hamilton PB, Douglas MSV, Smol JP. Diatoms of North America: the freshwater floras of Prince Patrick, Ellef Ringnes and northern Ellesmere Islands from the Canadian Arctic Archipelago Iconographia Diatomologica vol. 17 Koenigstein: Koeltz Scientific Books; 2008.

[41] Poulin M, Massé G, Belt ST, Delavault P, Rousseau F, Robert JM, et al Morphological, biochemical and molecular evidence for the transfer of *Gyrosigma nipkowii* Meister to the genus *Haslea* (Bacillariophyta). Eur J Phycol. 2004;39: 181–195.

[42] Lynch ED, Lee MK, Morrow JE, Welcsh PL, León PE, King MC. Nonsyndromic Deafness DFNA1 associated with mutation of a human homolog of the Drosophila gene diaphanous. Science. 1997;278: 1315–1318. 9360932

[43] Daugbjerg N, Andersen RA. A molecular phylogeny of the heterokont algae based on analyses of chloroplast-encoded *rbcL* sequence data. J Phycol. 1997;33: 1031–1041.

[44] Ludwig W, Strunk O, Westram R, Richter L, Meier H, Buchner A, et al ARB: a software environment for sequence data. Nucleic Acids Res. 2004;32: 1363–1371. doi: 10.1093/nar/gkh293 14985472 10.1093/nar/gkh293 PMC390282

[45] Tamura K, Peterson D, Peterson N, Stecher G, Nei M, Kumar S. MEGA5: molecular evolutionary genetics analysis using maximum likelihood, evolutionary distance, and maximum parsimony methods. Mol Biol Evol. 2011;28(10): 2731–2739. doi: 10.1093/molbev/msr121 21546353 10.1093/molbev/msr121 PMC3203626

[46] Stamatakis A. RAxML version 8: a tool for phylogenetic analysis and post-analysis of large phylogenies. Bioinformatics. 2014;30: 1312–1313. doi: 10.1093/bioinformatics/btu033 24451623 10.1093/bioinformatics/btu033 PMC3998144

[47] Jukes TH, Cantor CR. Evolution of protein molecules In: Mammalian protein metabolism (Ed. by Munro HN.). New York: Academic Press; 1969 pp. 21–132.

[48] Ribeiro LLCS. Intertidal benthic diatoms of the Tagus estuary: taxonomic composition and spatial-temporal variation. Thesis, Universidade de Lisboa. 2010. Available: http://repositorio.ul.pt/handle/10451/2330.

[49] Lang I, Kaczmarska I. A protocol for a single-cell PCR of diatoms from fixed samples: method validation using *Ditylum brightwellii* (T. West) Grunow. Diatom Res. 2011;26: 43–49.

[50] Mann DG, Vanormelingen P. An Inordinate Fondness? The Number, Distributions, and Origins of Diatom Species. J Eukaryot Microbiol. 2013;60: 414–420. doi: 10.1111/jeu.12047 23710621 10.1111/jeu.12047

[51] Bruder K, Medlin LK. Morphological and molecular investigations of naviculoid diatoms. II. Selected genera and families. Diatom Res. 2008;23: 283–329.

[52] Felsenstein J. Cases in which parsimony or compatibility methods will be positively misleading. Syst Biol. 1978;27: 401–410.

[53] MacGillivary ML, Kaczmarska I. Survey of the efficacy of a short fragment of the *rbcL* gene as a supplemental DNA barcode for diatoms. J Eukaryot Microbiol. 2011;58: 529–536. doi: 10.1111/j.1550-7408.2011.00585.x 22092527 10.1111/j.1550-7408.2011.00585.x

[54] Hamsher SE, Evans KM, Mann DG, Poulíčková A, Saunders GW. Barcoding diatoms: exploring alternatives to COI-5P. Protist. 2011;162: 405–422. doi: 10.1016/j.protis.2010.09.005 21239228 10.1016/j.protis.2010.09.005

[55] Kermarrec L, Franc A, Rimet F, Chaumeil P, Frigerio JM, Humbert JF, et al A next-generation sequencing approach to river biomonitoring using benthic diatoms. Freshw Sci. 2014;33: 349–363.

